# The effect of aggressive group norms on young adults’ conformity behavior in WhatsApp chats: a vignette-based experiment

**DOI:** 10.1038/s41598-024-67915-9

**Published:** 2024-07-26

**Authors:** Annika Kreuder, Ulrich Frick, Jennifer Klütsch, Luise Haehn, Sabine J. Schlittmeier

**Affiliations:** 1 Work and Engineering Psychology, RWTH Aachen University, Aachen, Germany; 2 HSD Hochschule Döpfer, Cologne, Germany; 3 Institute for Clinical Epidemiology and Biometry, Julius-Maximilians-University Würzburg, Würzburg, Germany

**Keywords:** Human behaviour, Psychology

## Abstract

Social networking and messaging applications, such as WhatsApp, have become an essential social environment for adolescents and young adults (AYA). While facilitating connectivity, they also bear hazards, including cyber-aggression. This study investigates the impact of (aggressive) group norms on AYA's propensity to expect cyberaggressive behaviors within different group chats. Based on a vignette scenario, realistically simulated WhatsApp group chats enabled scrutinizing, if and how exemplary reactions (funny, aggressive, friendly) of group members influence AYA's conformity to cyber-aggression (*N* = 500, aged 16 to 29). Additionally, we examined the effect of chat group type—close friends versus fellow students—on the anticipation of aggressive reactions. Sociodemographic, social, and developmental-psychological factors were evaluated for potential effects. Multilevel logistic regression analyses indicated that aggressive group norms significantly predict cyber-aggression anticipation, while no effect of chat group type was observed. Controlling for the size and vivacity of participant’s friend group, gender, age, and educational status were significant predictors: males, younger participants, and non-university students expected higher levels of cyber-aggression conformity. This study underlines the importance of group dynamics on perceptions of cyber-aggression and hints at individual risk factors for AYA's digital communication behavior.

## Introduction

WhatsApp's pervasive presence in the lives of adolescents and young adults (AYA) offers a unique lens through which to examine the social dynamics of cyber-aggression. Social norms and related conformity influence responses to cyber-aggressive behavior within WhatsApp groups. These effects are shaped by age-specific psychological factors and sociodemographic characteristics like gender and age, as well as group composition, distinguishing between friends and fellow students. This study tests these influencing factors, examining AYAs’ prediction of their close friends’ digital communication behaviors, to offer insights into interventions that may reduce cyber-aggression and promote healthier digital interaction among young users.

Social networks and mobile messaging applications have become integral to the everyday interactions of AYA^1,2^. WhatsApp, as one of the best-known applications, enjoys particular popularity among AYA with 89% of 16 to 29-year-olds using this platform in Germany^3^. While facilitating connectivity, mobile messaging apps also present challenges, including the risk of cyber-aggression among users^2,4,5^. Cyber-aggression manifests through harmful and offensive behaviors such as the sharing of images without consent, impersonation, and derogatory comments^6–8^. While cyberbullying is defined as repeated negative behavior between peers, cyber-aggression includes incidental and non-repetitive behaviors, which can still have a harmful impact^9,10^. This could involve, for instance, circulating a private photo within a group chat, leading to detrimental effects on the emotional well-being of young people^11^.

WhatsApp, with its group chat feature, has been observed as a common space for such group-oriented aggressive behavior^4,11^. The implications are concerning due to the online environment's expansive reach, potential for perpetuity, and the public nature of shared information. Given that approximately 30% of young WhatsApp users report experiencing cyber-aggression, understanding these social processes is crucial for prevention and intervention^11,12^. Consequently, in our current study, we aim to replicate and build upon previous findings that have examined the relationship between social norms and engagement in cyber-aggression.

Understanding the social and individual influences on cyberbullying behavior has been the focus of many studies. Starting with social influences, peer group influences in particular are considered to be important in promoting cyberbullying and cyber-aggression^5,13^ and shape adolescents' behaviors, beliefs, and attitudes by aligning them with group expectations and norms^14–16^. The Social Identity (SI) perspective^17,18^, which focuses on how group norms, social identification, and the desire for positive group distinctiveness influence individual behavior, helps to clarify how individuals' actions are shaped by their membership in particular groups and their desire to maintain a positive image within those groups.

Previously, and first applied to messaging app contexts by Bleize et al.^9,19^, social identification (self-investment and centrality) significantly predicted overall conformity in WhatsApp groups. Norms, which typically refer to implicit shared rules or values about appropriate, expected, or desirable attitudes and behaviors in social groups^20^, further guide group members' behaviors and beliefs, according to Hogg and Reid^15^. Conformity to these peer group norms varies by group characteristics, influencing the prevalence of cyberbullying within different groups^21,22^. Studies of cyber-aggression specifically highlight the role of disrespectful, rude, and aggressive norms in predicting engagement in such behaviors^5,13,23^. These norms serve as indicators of actual aggressive acts or predictions, which are otherwise challenging to measure. Consistent with previous research^24,25^, Bleize et al. found that social identification with WhatsApp group members was generally positively associated with general group conformity on WhatsApp^19^. Specifically for conformity to cyber-aggression, findings on social identification and the subjective importance of a social group (group centrality) were more equivocal^9,19^. Therefore, researchers suggest using more realistic WhatsApp settings in experimental studies to better understand conformity effects^19^. As an initial gap to be addressed, this study will use a novel approach by simulating realistic aggressive WhatsApp conversations in a vignette-based experiment.

Moving on to psychological and sociodemographic variables, research has identified variables such as impulsivity, empathy, school aggression, and being a victim of cyberbullying as key predictors of cyberbullying perpetration among adolescents^26,27^. Gender differences have been extensively studied, but the results remain inconsistent^4^. Some researchers suggest that males are more likely to be the aggressors^24,28^, while Khurana et al. propose that females may be more aggressive in online settings^29^. Álvarez-Garcia et al. suggested that gender may have indirect influences on such behaviors, a position that is not universally accepted^28–31^.

Researchers agree that younger people are particularly vulnerable to cyber victimization and perpetration. However, the exact nature of this relationship is still debated. Aizenkot et al. report a decrease in cyberbullying victimization in WhatsApp classmate groups from elementary to high school^4^. In contrast, specific behaviors such as posting offensive photos without consent, increased from elementary to high school^4^. A study focusing on cyberbullying among adults found that young adults aged 18–25 years experienced the highest levels of lifetime and past-month cyberbullying^32^. Buelga et al. suggest a quadratic trend, with perpetration of cyber-aggression increasing from 11–17 years to 18–26 years and then decreasing^33^. Especially older adolescents over the age of 15 appear to be at an increased risk of engaging in cyberbullying^26,34^. Surprisingly, although evidence suggests cyber victimization and perpetration peak in older adolescence and remain elevated into the early twenties^35^, most research on cyber-aggression has focused on its prevalence, risk factors, and outcomes among children and early adolescents^5,26,30^. Therefore, as a second research gap to be addressed, this study will focus on older adolescents and young adults (AYA; 16 to 29 years^36^).

Given the significant role of social influence in cyber-aggression, it is not surprising that group-based cyber-aggression is most prevalent among this age group. AYA are in a critical period of development characterized by significant physical, cognitive, emotional and social changes^36–39^. These include increased risk-taking behavior and a heightened sensitivity to social influences^39^. As adolescents mature, they become more aware of social norms, including social cues such as emoticons^40^. These signals may influence their interpretation of others' intentions in digital communication. Consistent with the dual systems model^41^ that highlights the relationship between adolescents’ immature neurocognitive development and related behaviors^38,39^, there is some evidence that strong perceptions of social cues and general social motivations in mid to late adolescence may override cognitive controls, potentially leading to increased conformity and, in some cases, aggressive behaviors^40^.

In addition to undergoing neurocognitive changes, AYA also face important decisions about their educational and career paths in order to achieve various developmental milestones that mark their transition to adulthood^37^. According to Havighurst’s Developmental Task Theory^37^, these tasks also include forming committed relationships, and establishing peer group connections, which are essential for social development and emotional maturity as outlined in the model and supported by numerous surveys and studies^42–45^. The Internet plays a central role in AYAs’ developmental task fulfillment, supporting identity exploration, autonomy, and the establishment of peer relationships^46^. Despite its importance to this age group, research has been scarce, with little focus on developmental patterns and psychosocial factors^47,48^. As a result, little is known about how and why cyber-aggression peaks during adolescence and young adulthood.

In summary, although many studies have examined the influence of social norms on adolescents' online behavior, research on the psychological underpinnings and contributing factors of cyber-aggression in messaging apps, especially among AYA, is still limited, mostly correlational, and thus not suitable for establishing cause-and-effect relationships. The series of studies by Bleize et al. from 2021 to 2022 is an exception to the limited literature on conformity to cyber-aggression in adolescents on WhatsApp^9,12,19^. To further address this research gap, we employed a novel approach with experimental rigor. To examine AYAs’ conformity to group chat norms, particularly aggressive norms, we simulated realistic WhatsApp conversations, in which we chose a combination of visual and written-verbal cyber-aggressive behavior^8^. Our study experimentally tests whether AYA in WhatsApp groups adhere to (aggressive) norms, as evidenced by the displayed reactions of group members, when an offensive photo is posted without consent. Consistent with previous research on group norms^9,19^, we hypothesize that:

### H_1_

Adolescents and young adults may conform to aggressive group norms in WhatsApp group settings. They tend to expect more cyber-aggressive responses when aggressive reactions are already present in a group chat.

In addition, we will explore whether the type of group chat, specifically when the group consists of friends rather than fellow students, influences conformity behavior. Previous studies have shown that adolescents are more likely to conform to aggressive behavior exhibited by close peers rather than distant peers^5,49,50^. Therefore, in contrast to the recent findings of Bleize et al.^9^, who did not find similar group effects on conformity due to small effect sizes, we hypothesize that:

### H_2_

Adolescents and young adults are more likely to conform to aggressive group norms in WhatsApp groups, particularly when the group consists of close friends rather than peers from educational settings.

Furthermore, given that previous findings have been inconsistent^24–29^, our study aims to examine whether and how sociodemographic factors such as age, gender, and educational status, in addition to psychological determinants such as developmental status, influence expected conformity to cyber-aggressive norms. This area of research, particularly regarding the AYA age group and our developmental approach, is new and exploratory.

## Methods

This study utilized a vignette-based online study presenting three different examples of group chat conversations as stimuli, which were designed to induce various styles of answering modes (forms and tones of responses). Participants estimated the number of their close friends who would respond to the messages in a specific form (emoticons, comments) and tone (funny, aggressive, or friendly), which served as the dependent variable. AYAs’ (16–29 years old) conformity to cyber-aggressive norms could thus be tested. The study was part of a larger research project (A-DigiKomp), which focused on young people’s digital competences. It was approved by the ethics committee of HSD Hochschule Döpfer without obligations (decision BEth_20_22 dated May 19, 2022). All methods were performed in accordance with the 1964 Helsinki Declaration and its subsequent revisions.

### Sample

We recruited 553 initial participants through various digital channels (mailing lists, social media) and direct contacts at German educational institutions. Referring to AYA^36^, we excluded participants over 29 years of age and those who did not pass the control items (*n*_excluded_ = 53, 9.6%). The final sample consisted of *N* = 500 individuals (398 females, 99 males, 3 no indication, 0 non-binary) with a mean age of *M* = 22.28 years (*SD* = 2.55, Range = 16–29 years). Most participants were university students (80.0%), followed by those in the labor force (9.8%), apprentices (7.8%) and school students (2.4%). Educational attainment was high, with most having A-Levels (67.8%) or university degrees (22.4%).

All participants gave their informed consent prior to data collection. Participation was voluntary, and participants could win vouchers or earn course credits (university students of RWTH Aachen University and HSD Hochschule Döpfer) upon completion of the study.

### Procedure and experimental manipulation

The study was conducted online from July to November 2022. Participants first answered introductory questions about their sociodemographic data and daily usage of the Internet, social media, and instant messaging apps. Without prior disclosure of the research topic, participants were then presented with image-based vignette scenarios (experimental manipulation) and questionnaires (concomitant variables) to assess conformity to cyber-aggression in WhatsApp group chats.

**Figure 1:**
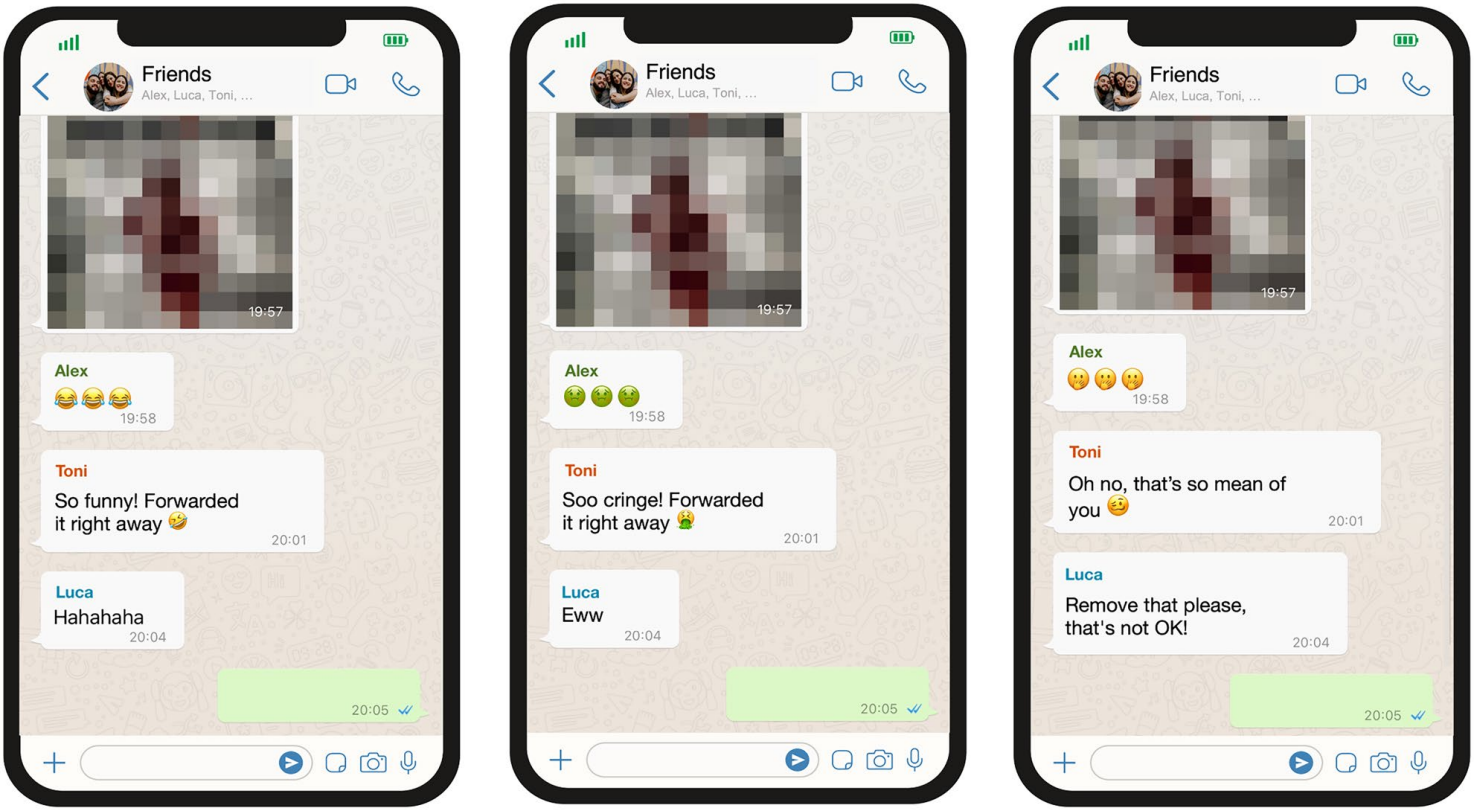
Examples of WhatsApp vignette scenarios with different group norms (from left to right: funny, aggressive, friendly). Participants were either presented with fictive group chats composed of friends (as displayed here) or fellow school/university students (as indicated in the WhatsApp chat group name). The pixelated photo is described as showing a person with slipped clothes while doing sports. The vignette scenarios have been translated from German into English.

We created six image-based vignettes by varying features of a group chat depicting a potential visual and written-verbal cyber-aggression scenario^8^. Participants were randomly assigned to either a friends’ or a fellow students’ WhatsApp chat condition. Both groups viewed three fictional group chat screenshots showing a pixelated individual’s photo (see Fig. 1). The chats depicted three group norms: (1) funny/mocking, (2) insulting/aggressive, and (3) compassionate/friendly, with manipulated reaction tones and forms (emoticons, comments) while keeping other characteristics constant. Gender-neutral names were used for the group members, and the presentation order was randomized to minimize confounding or sequencing effects.

Participants were instructed to imagine that the shared image depicted an external person in an unfavorable pose without consent, using a schematic representation for ethical reasons (see Fig. 1). They completed two tasks: estimating the number of close friends they contact via social media (control variable) and estimating how many of those friends would respond with specific reactions (funny, aggressive, or friendly) for each chat scenario.

After completing these tasks, participants answered questionnaires assessing personal characteristics, including *developmental task fulfillment* and *importance* (26 items extended version)^51^, *ingroup prototypicality*, and *ingroup identification* (2 items)^5,52,53^. To ensure attentive participation, two control items were included. Participants could optionally provide feedback on the study. The debriefing phase provided information about the study's purpose. The study took approximately 30 min to administer. Data collection was executed via PsychoPy, enhanced with HTML and JavaScript, and hosted on Pavlovia (https://pavlovia.org/).

### Measures

#### Outcome variable ‘cyber-aggression’

We operationalized cyber-aggression on WhatsApp as expected conformity to aggressive group norms (reactions in the chat history). To ask respondents directly about their own 'cyber-aggressive’ behavior is subject to strong effects of social desirability. At least, researchers of a survey like ours would be perceived as protagonists of a norm against offensive Internet behavior. Answers can therefore be expected to be edited in favor of a societal norm of ‘netiquette’ in some way. On the contrary, in case our respondents share a self-categorization as ‘bad boy’/’bad girl’ for some reason (not uncommon among AYA), reactions might be biased and exaggerated in a direction of social deviance. We therefore used an indirect measure (similar to the contrast vignette technique of Burstin et al.^54^) by presenting a specific situation and then asking the respondents to estimate the number of their close friends who they expect to behave in a certain way. The instructions provided were as follows (English translation):

*You see this chat history in a WhatsApp group with your friends [fellow students]. In this group, a photo of a person who is not part of the group has just been shared without consent. The photo shows the person with slipped clothes while doing sports. Please estimate the number of your close friends with whom you are in contact via smartphone (e.g. via WhatsApp) and/or social networks (e.g. Instagram, TikTok, Snapchat, Twitter). ______*

*Now please think about your close friends with whom you are in contact via smartphone. In the situation shown, how many of your friends would react to the photo in the chat in some way if they were in the WhatsApp group?*

After indicating the number of close friends, participants were asked to estimate the number of their close friends who would react to the messages in any of the following nine ways: **(1) forward the photo without consent**, (2) send a laughing emoji (face that laughs tears), (3) write a ‘funny’ comment, **(4) send an emoji expressing disgust/aversion (nauseated face)**, **(5) write an insulting/aggressive comment**, (6) send an emoji expressing to be shocked (face with open eyes and hand over mouth), (7) write a comment expressing compassion, (8) write a comment calling for the photo to be deleted, (9) show a different reaction (open input). Response options (1), (4) and (5) were classified as cyber-aggressive reactions. Multiple answers for each reaction were possible but not mandatory. Responses indicating any friends exhibiting aggressive reactions were subsequently simplified and categorized as ‘cyber-aggression expected’ vs. ‘no cyber-aggression expected’.

#### Concomitant variables/additional measures

Sociodemographic variables and Internet use were measured as potential influencing variables. The participants' gender (female, male, non-binary, no indication), age, and educational status (high school, university, work, etc.) were queried. Participants were asked to report the number of hours they spend online daily for personal purposes, such as communicating with friends or entertainment, and for educational or work-related activities, such as attending online meetings or researching information for assignments. Additionally, they were asked to specify the instant messaging services and social media platforms they use regularly for communication.

Inspired by the Developmental Task Questionnaire for Young Adults (DTQ-YA) by Seiffge-Krenke^51^, a list of 13 items asking to what extent the respondent would rate a particular developmental task (e.g., entering the workforce, being part of a peer group, maintaining a stable relationship) as accomplished (*‘I have not yet achieved’, ‘I have begun’, ‘I have already achieved’*), and how much they would rate the same list as important *(‘not important to me’, ‘somewhat important to me’, ‘very important to me’)*. Based on focus group discussions^55^ and literature research, the questionnaire was adapted to the current state of the art and the reality of AYAs’ lives nowadays^46,42^.The resulting scales reached a Cronbach’s alpha of α = 0.57 (fulfillment) and α = 0.61 (importance), indicating only limited reliability.

A single item was used to measure self-perceived ingroup prototypicality among friends who communicated through instant messages, email, and social networks (see Piccoli et al.^5^ and Jetten et al.^49^). Participants were asked to rate their typicality within the group on a 4-point scale from 1 *‘not typical at all’* to 4 *'very typical'.* Higher scores on this scale indicate a higher level of perceived typicality within the group.

Cognitive identification with the ingroup was measured using the single item ‘Inclusion of the Ingroup in the Self (IIS)’ (7 point scale, *no overlap* to *almost complete overlap*) proposed by Tropp and Wright^53^ and also used by Piccoli et al.^5^. Details of construction and application can be found there. Higher scores on this scale indicate a greater level of closeness with friends who are in contact through smartphones and the Internet.

In addition, two control variables were measured to account for potential confounding influences related to the operationalization of cyber-aggression. The first control variable was 'number of close friends', measured by asking participants to estimate the number of close friends they contact via smartphone or social networks.

The second control variable was 'expected group vivacity', measured by the total number of expected reactions from close friends for each scenario. Group vivacity reflects the collective energy, enthusiasm, and level of interaction within the group and can be operationalized through indicators such as the frequency and intensity of interactions or the presence of discussions. Controlling for the size of one's close friend group and the general expected liveliness of WhatsApp group chats was critical, as both factors may influence AYA's estimates of the number of their friends who would potentially engage in cyber-aggression.

All items were originally presented in German and have been translated into English for the purposes of this paper.

### Design and statistical analyses

The experiment used a 2 × 3 factorial design with *type of group chat* (friend group vs. fellow student group) as a between-subjects factor and *displayed group norm* (funny/mocking, insulting/aggressive, compassionate/friendly) as a within-subject factor. The dependent variable was expected *cyber-aggressive reaction* (0/1). For reasons of simplicity, and because answers were given in a clear zero-inflated distribution (a considerable part of participants would not expect any of their friends to behave aggressive), the dependent variable of the experiment was summed up over the three indicative options as a dichotomized variable ‘cyber-aggression expected as present/not present’ (1/0) in the defined type of group (see above for detailed information).

During the confirmatory regression analyses (multilevel logistic regression), we initially tested the experimentally varied factors: *type of group chat* (level 2) and *displayed* *group norm* (level 1). This stage focused on establishing the causal effects of the experimental manipulations without the influence of additional variables. The confirmatory model aimed to provide clear, causally interpretable results regarding the hypotheses *H*_*1*_ and *H*_*2*_ of our study.

Following this, we adopted a hierarchical regression approach to build a comprehensive model incorporating various concomitant variables^56–58^. We dichotomized all concomitant variables into 0/1 categories based on median splits to simplify the analysis and enhance interpretability (see Table 1 for median values and Table 3 for references)^59^. The hierarchical model was constructed in several stages, each adding a new set of variables to the confirmatory model to assess their potential impact and improve model fit. The order in which the exploratory factors were added was based on a theory-driven approach^57,58^, incorporating predictors that were already presumed to yield anticipated outcomes first and more exploratory ones last. In the first step, we added the *number of close friends* and *group vivacity* as control variables to hold them constant. Next, we added sociodemographic variables (*age, gender, education level*), social identification variables (*ingroup prototypicality*, and *ingroup identification*), *intensity of Internet usage* (personal and work-related), and *developmental task fulfillment* and *importance*. Model improvement was subsequently tested by comparing log-likelihood ratios, Akaike Information Criterion (AIC) and the Bayesian Information Criterion (BIC) between each step.

**Table 1:** Descriptive statistics of the sample.

	*N*	Mean	*SD*	Min	1st Qu.	Median	3rd Qu.	Max
Control variables								
Number of close friends	493	9.66	7.48	0.00	5.00	7.00	10.00	50.00
Number of anticipated reactions^a^	500	11.75	13.21	0.00	5.00	8.00	15.00	133.00
Sociodemographic								
Age	500	22.28	2.55	16.00	20.00	22.00	24.25	29.00
Social identification								
Ingroup prototypicality	500	2.89	0.76	1.00	2.00	3.00	3.00	4.00
Ingroup identification	500	4.58	1.39	1.00	4.00	5.00	6.00	7.00
Internet usage (hours/day)								
Personal use	500	3.84	1.77	0.64	2.57	3.57	4.57	12.14
Work/education	500	4.04	2.21	0.00	2.42	3.71	5.43	17.14
Developmental tasks								
Fulfillment	500	13.77	0.49	0.00	11.00	14.00	17.00	23.00
Importance	500	19.63	3.18	0.00	18.00	20.00	22.00	26.00

^a^Group vivacity.

Finally, a parsimonious prediction model was computed, retaining only those parameters which inclusion in the model significantly improved the model fit. This three-step approach, beginning with a confirmatory model, followed by a hierarchical inclusion of concomitant variables, and culminating in a refined final model, allowed us to systematically examine the contribution of various factors to friends' predicted cyber-aggression. The statistical analyses were performed using R^60^ and RStudio (Version: 2022.12.10)^61^, especially the lme4 package (v1.1.27.1)^62^. Significance tests were performed at α = 0.05.

## Results

### Preparatory analyses

Descriptive analyses showed that all participants used instant messaging applications, with WhatsApp being the most popular (97.8%). Participants spent an average of four hours per day online for personal use (*M* = 3.84, *SD* = 1.77) and for work/education (*M* = 4.04, *SD* = 2.21). On average, they had completed or were completing 9 out of 13 developmental tasks, with 12 tasks rated as *somewhat* or *very important* (see Table 1). Participants reported having an average of ten close friends (*M* = 9.66, *SD* = 7.48) and estimated 12 reactions (*M* = 11.75, *SD* = 13.48) from these friends as group vivacity (e.g., six comments, six emoticons). The median values of the predictor variables are shown in Table 1.

Of the 500 participants, 259 were assigned to the friend group condition (51.8%) and 241 to the fellow student group condition (48.2%). Randomization checks revealed no significant differences between groups in terms of age, gender, education level, Internet use, developmental tasks, number of close friends, or expected group vivacity (*p* > 0.05). However, participants in the friend group condition spent significantly less time online in their free time than those in the fellow student group condition (36 min less per day; *t*(498) = 4.45, *p* < 0.05, Cohen's *d* = 0.23).

### Multilevel logistic regression

The chance for cyber-aggression reactions (log-odds) was quantified through multilevel logistic regression analysis. The intra-class correlation coefficient (ICC) indicated that approximately 76% of the variance in cyber-aggression can be attributed to inter-individual differences. Out of all 1,500 trials reported, 672 (44.7%) could be rated as cyber-aggressive. This means that in 44.7% of all observations (level 1), participants expected at least one of their friends to engage in some form of cyber-aggression (e.g., aggressive comment/emoticon). Taking repeated measures into account, this means that 301 participants (59.9%) anticipated some form of aggressive reaction in at least one of the given scenarios at the subject level (level 2).

#### Confirmatory analysis of cyber-aggressive reactions

We conducted a confirmatory analysis using multilevel logistic regression, clustered by participant (level 2), and predicted by group chat type (level 2) and group norm (level 1). Results from Table 2 indicate that an aggressive group norm in the chat significantly predicted cyber-aggression expectations. Participants anticipated a greater amount of cyber-aggressive reactions when aggressive messages were already displayed (β = 0.54, OR = 1.72, 95% CI [1.16–2.54],* p* < 0.001). The odds of estimating an aggressive response increased by 72% in the presence of a cyber-aggressive norm, compared to a friendly or funny norm. Conversely, the implementation of a funny group norm did not result in a significant increase in cyber-aggression (ß = 0.21, 95% CI [0.84–1.83], *p* = 0.28). Contrary to our initial hypothesis, we did not observe an effect of group chat type (friends vs. fellow students) on cyber-aggression (ß = − 0.40, 95% CI [0.33–0.98], *p* = 0.67).

**Table 2:** Results of the confirmatory model for cyber-aggressive reactions as predicted by type of group chat and group norm.

Predictors	Coefficient	Wald χ^2^	*p*	OR	95% CI
(Intercept)	− 0.59	4.07	< 0.01	0.56	0.31–0.98
Type of group chat (friends)^a^	− 0.40	1.18	0.28	0.67	0.33–1.38
Group norm (funny)^b^	0.21	1.18	0.28	1.24	0.84–1.83
Group norm (aggressive)^b^	0.54	7.42	< 0.001	1.72	1.16–2.54
Random effects					
ICC	0.76				
Observations (Level 1)	1500				
* N* (Level 2)	500				
Marginal R^2^/conditional R^2^	0.006/0.763				

Reference categories: ^a^fellow student group, ^b^friendly group norm.

#### Exploratory analysis including concomitant variables

**Figure 2:**
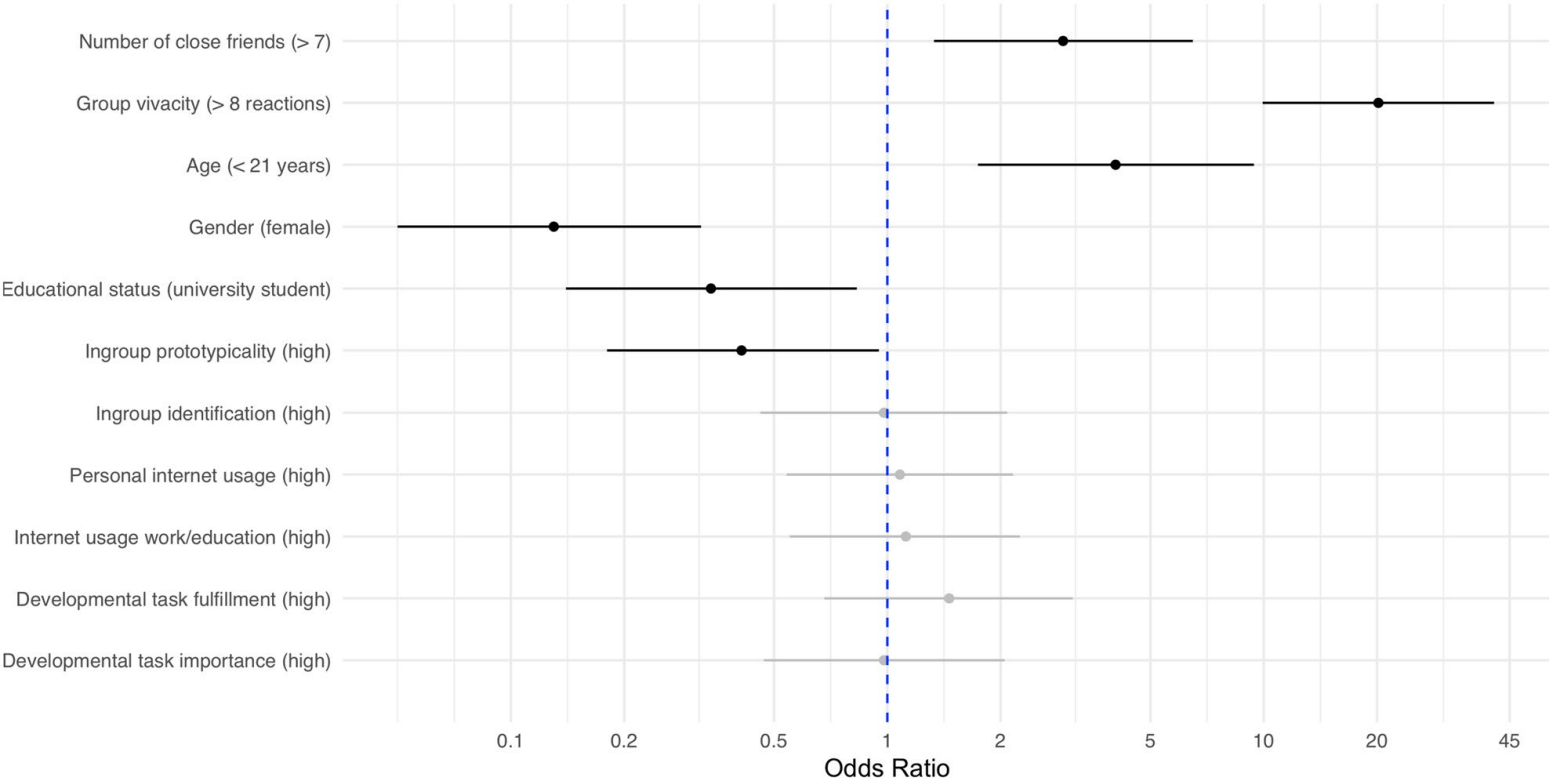
Exploratory predictors in the multiple logistic regression of expected cyber-aggression. The forest plot displays odds ratios and 95% confidence intervals on a log-scale. Significant predictors are indicated by black lines, while non-significant predictors are indicated by grey lines. Reference categories are indexed in Table 3.

The initial step of the hierarchical multiple logistic regression was conducted to determine the influence of the control variables, number of close friends and group vivacity, on the prediction of cyber-aggression. This model showed a significantly better fit than the confirmatory model, χ^2^(2) = 125.27,* p* < 0.001, ΔAIC = − 121.3. In a second step, sociodemographic variables (age, gender, education level) were added to the model. This addition significantly improved the model fit, χ^2^(3) = 34.50, *p* < 0.001, ΔAIC = − 28.5. The third step included social identification variables (ingroup prototypicality, ingroup identification). Although this step did not significantly improve the model, χ^2^(2) = 4.64, *p* = 0.10, ΔAIC = − 0.6, it slightly increased the marginal R^2^ to 0.250 and the conditional R^2^ to 0.805. In the fourth step, variables for the intensity of Internet use were included. This step did not significantly enhance the model, χ^2^(2) = 0.17, *p* = 0.92, ΔAIC =  + 3.8. In the final step, adding the AYA developmental task fulfillment and importance variables, did not significantly improve predictive performance, χ^2^(2) = 0.96, *p* = 0.62, ΔAIC =  + 3.1. In conclusion, the hierarchical model-building process identified key predictors of cyber-aggression that contributed to the model's predictive performance, as detailed in Table 3 and Fig. 2.

**Table 3:** Hierarchical multiple logistic regression of expected cyber-aggressive reactions as predicted by type of group chat and group norm.

Model	Model test	AIC	Δ AIC	R^2^_Marginal_	R^2^_Conditional_	*p*
1: Size and vivacity of friends group	χ^2^(2) = 125.27	1528.2	− 121.3	0.189	0.797	< 0.001
2: Sociodemographics	χ^2^(3) = 34.50	1499.7	− 28.5	0.243	0.803	< 0.001
3: Social identification	χ^2^(2) = 4.64	1499.1	− 0.6	0.250	0.805	0.10
4: Internet usage	χ^2^(2) = 0.17	1502.9	+ 3.8	0.251	0.805	0.92
5: Developmental tasks (full model)	χ^2^(2) = 0.96	1506.0	+ 3.1	0.252	0.805	0.62

Model parameter	Coefficient	Wald χ^2^	OR	95% CI	*p*	
5: Full model						
(Intercept)	0.02	0.00	0.98	0.24–3.99	0.97	
Type of group chat (friends)^a^	− 0.30	0.70	0.74	0.37–1.49	0.40	
Group norm (funny)^b^	0.18	0.71	1.19	0.79–1.79	0.40	
Group norm (aggressive)^b^	0.60	8.14	1.81	1.21–2.74	< 0.01	
Number of close friends (> 7)^c^	1.08	7.12	2.93	1.33–6.48	< 0.01	
Group vivacity (> 8 reactions)^d^	3.00	69.14	20.16	9.93–40.92	< 0.001	
Age (< 21 years)^e^	1.40	10.48	4.04	1.74–9.42	< 0.001	
Gender (female)^f^	− 2.06	18.84	0.13	0.05–0.32	< 0.001	
Educational status (university student)^g^	− 1.08	5.59	0.34	0.14–0.83	< 0.05	
Ingroup prototypicality (high)^h^	− 0.90	4.29	0.41	0.18–0.95	< 0.05	
Ingroup identification (high)^i^	− 0.02	0.00	0.98	0.46–2.08	0.96	
Personal Internet usage (high)^j^	0.07	0.04	1.08	0.54–2.16	0.84	
Internet usage work/education (high)^j^	0.11	0.10	1.12	0.55–2.25	0.76	
Developmental task fulfillment (high)^k^	0.38	0.94	1.46	0.68–3.11	0.33	
Developmental task importance (high)^l^	− 0.02	0.00	0.98	0.47–2.05	0.96	

Predictors in steps 1–4 have been omitted for the sake of clarity. Model improvement was tested by comparing the log-likelihood ratios between each step. Reference categories: ^a^fellow student group, ^b^friendly group norm, ^c^0–7 close friends, ^d^lower anticipated vivacity (> 8 group reactions), ^e^21–29 years old, ^f^male respondents, ^g^all other educational level, ^h^ingroup prototypicality low, ^i^ingroup identification low, ^j^lower level of Internet usage (median or below), ^k^lower fulfillment of developmental tasks (median or below), ^l^lower importance of developmental tasks (median or below).

#### Final parsimonious model

The final parsimonious regression model, which was built using only variable sets from the exploratory hierarchical model that significantly improved the model's predictive performance, revealed several relationships between the studied predictors and cyber-aggressive reactions (see Table 4). The intercept of the model indicated a baseline likelihood of cyber-aggression when all predictors were at their reference levels (OR = 0.81, 95% CI [0.25, 2.66], *p* = 0.73). Comparing friend groups to fellow student groups did not significantly predict aggressive reactions (OR = 0.69, 95% CI [0.34, 1.37], *p* = 0.28), suggesting that group chat type alone does not influence the propensity for cyber-aggression in the present study. Group norms were found to be a significant factor, with aggressive norms increasing the likelihood of cyber-aggression by 1.82 compared to friendly or funny norms (OR = 1.82, 95% CI [1.21, 2.73], *p* < 0.01).

**Table 4:** Final parsimonious logistic regression model for cyber-aggressive reactions as predicted by type of group chat and group norm.

Predictors	Coefficient	Wald χ^2^	*p*	OR	95% CI
(Intercept)	− 0.21	0.12	0.73	0.81	0.25–2.66
Type of group chat (friends)^a^	− 0.38	1.15	0.28	0.69	0.34–1.37
Group norm (funny)^b^	0.18	0.71	0.40	1.19	0.79–1.79
Group norm (aggressive)^b^	0.60	8.21	< 0.01	1.82	1.21–2.73
Number of close friends (> 7)^c^	1.04	6.88	< 0.01	2.82	1.30–6.11
Group vivacity (> 8 reactions)^d^	2.97	69.94	< 0.001	19.56	9.74–39.27
Age (< 21 years)^e^	1.20	8.85	< 0.01	3.33	1.51–7.36
Gender (female)^f^	− 2.13	21.05	< 0.001	0.12	0.05–0.30
Educational status (university student)^g^	− 1.14	6.53	< 0.05	0.32	0.13–0.77
Random effects					
ICC	0.76				
Observations (Level 1)	1500				
* N* (Level 2)	500				
Marginal R^2^/conditional R^2^	0.243/0.803				

Predictive performance and parsimony of the final model were preferred over the statistical significance of individual predictors. Reference categories: ^a^fellow student group, ^b^friendly group norm, ^c^0–7 close friends, ^d^lower anticipated vivacity (> 8 group reactions), ^e^21–29 years old, ^f^male respondents, ^g^all other educational level, ^h^ingroup prototypicality low, ^i^ingroup identification low, ^j^lower level of Internet usage (median or below), ^k^lower fulfillment of developmental tasks (median or below), ^l^lower importance of developmental tasks (median or below).

The likelihood of cyber-aggression was found to be higher for individuals with more than seven close friends (OR = 2.82, 95% CI [1.30, 6.11], *p* < 0.01). A high level of group vivacity, as indicated by anticipating more than eight WhatsApp group reactions per chat, was found to be significantly associated with cyber-aggression (OR = 19.56, 95% CI [9.74, 39.27], *p* < 0.001). After controlling for both control variables, it was found that individuals under the age of 21 were significantly more likely to anticipate cyber-aggression from their friends than those aged 21 to 29 (OR = 3.33, 95% CI [1.51, 7.36], *p* < 0.01). Female respondents were 0.12 times less likely than males to anticipate cyber-aggression in their WhatsApp groups (OR = 0.12, 95% CI [0.05, 0.30], *p* < 0.001). Furthermore, university students showed a negative association with expecting cyber-aggression compared to respondents with other educational statuses (OR = 0.32, 95% CI [0.13, 0.77], *p* < 0.05).

The final model's marginal R^2^ of 0.243 and conditional R^2^ of 0.803 indicate that the predictors accounted for a substantial proportion of the variance in aggressive reactions. A likelihood-ratio test showed a significant improvement in model fit after the variable sets were added (χ^2^(5) = 159.77, *p* < 0.0001). The AIC and BIC decreased from 1649.5 to 1499.7 and from 1676.1 to 1552.9, respectively, indicating an improved fit. The full model, including additional predictors, did not improve model fit (χ^2^(6) = 5.77, *p* = 0.45) and had higher AIC and BIC values (1506.0 and 1591.0). This suggests that the final model, including the control and concomitant variables of interest, not only fits the data better than the confirmatory model, but is also more parsimonious.

## Discussion

Our study explored expected cyber-aggressive reactions of 16–29-year-olds in WhatsApp group chats when responding to an offensive photo shared without consent, and examined whether induced group norms—funny, aggressive, or friendly—influenced expected conformity to aggression. We also manipulated and evaluated chat group type (close friends versus fellow students). To examine the association between sociodemographic variables, social identification variables, developmental tasks, and cyber-aggression and to control for the size and vivacity of friend groups, we conducted an exploratory and extended version of the confirmatory analysis model. Our results therefore provide some insight into the influence of groups on cognition and emotion.

In line with our first hypothesis, the induction of different group norms (fictitious first reactions in the respective chat, see Fig. 1) affected the conformity to cyber-aggression as tolerable behavior: AYA expected significantly more aggressive reactions from close peers in their group chats when aggressive reactions were already present. This effect remained stable even after adjusting for confounding variables (see Tables 3 and 4). The likelihood of cyber-aggressive responses was almost twice as high as in the other group norm conditions (friendly, funny). This result supports the findings of other researchers^5,19^ who examined cyber-aggression in early adolescents and found a positive association between social identification and group conformity using observational approaches. We extend their findings by confirming the causal influence of induced group norms on the expected behavior of AYA in an experimental study. In the WhatsApp study that forms the basis of our approach, Bleize et al. first tested causal influences^9^, only finding a direct effect of accountability on conformity to aggressive norms in two experiments. Using a novel approach to simulate WhatsApp group chats, we were able to show that group norms—even experimentally induced ones—in the form of different interaction tones and reactions can have an impact on (the anticipation of) subsequent aggressive group behavior. This finding supports the view that cyber-aggression is highly peer-influenced^63,64^ and thus likely an age-, development-, and platform-related^2,4^ behavior.

Contrary to our second hypothesis, assigning respondents to different types of group chats, did not affect cyber-aggression conformity. Given our base rate of approximately 45% aggressive reactions and testing with a type I error risk of 0.05, our sample size allows us to detect an odds ratio of ≥ 1.35 with power > 0.8. This means that ‘type of group chat’ either has a smaller effect size or has no effect on the respondents’ cyber-aggressive tendencies (post hoc power calculations with G*Power 3.1)^65^. The latter explanation is supported by the fact that group type had no effect in the previously cited study by Bleize et al.^9^. The researchers manipulated group centrality, but this also failed to show a direct effect. However, both findings are inconsistent with what the Social Identity (SI) perspective^17,18^ and other previously proposed models^5,49,50^ predict. One possible interpretation could be that peer influence is so dominant among AYA in the WhatsApp environment that it suppresses the effect of different group importance. This may be particularly applicable for an AYA sample that tends to be highly susceptible to social influences in general^39,40^. Slagter et al.^50^ found that adolescents preferred certain attributes such as ‘coolness’, ‘acting mean’, and ‘higher peer status’ as a source of information within their social networks. Although beyond the focus of our study, future research could examine whether participants ascribed these traits to peers in the presented groups. If the non-significant ‘friends vs. fellow students’ result is the consequence of a relatively weak experimental manipulation, future studies could test a stronger one. For example, presenting WhatsApp group photos only to the friend group might contribute to a stronger differentiation between friends and fellow students and thus induce the expected effects. For now, it is assumed that individuals respond to group norms regardless of the strength of their social identification in the context of WhatsApp groups.

The predictive power of the statistical model was improved by the inclusion of potential confounding variables: age group, gender, educational status, as well as the control variables ‘number of close friends’ and ‘group vivacity’. However, due to their observational nature, they cannot be interpreted causally^66^. The successful inclusion of these variables reflects the existing literature, suggesting a substantial impact of demographic characteristics on cyber-aggression. First, the pronounced expectation of cyber-aggression among respondents under the age of 21, which is three times higher than that of their 21–29-year-old counterparts, aligns with studies indicating that emerging adults are more immersed in digital interactions, potentially increasing exposure to and engagement in cyber-aggression^26,32,34^.

Second, female participants were less likely to anticipate cyber-aggressive responses and exhibited less conformity in our experimental settings compared to males. This finding is consistent with other research on cyberbullying perpetration^5,25,28,67^. Therefore, gender differences can be explained by an interdisciplinary framework that incorporates gender role socialization theory^68,69^ and empirical research insights^53,70,71^. Gender role socialization theory explains that societal conditioning and deeply ingrained gender stereotypes result in different behavioral expectations and norms. During adolescence, males may be more likely to engage in deviant behaviors to gain peer approval. This tendency may escalate as gender roles become more pronounced and such behaviors are viewed as pathways to elevated status among male peers. In contrast, females are often associated with stereotypes of nurturance and emotional expressiveness, and therefore may be less likely to engage in such behaviors. Identity theory^72^ also supports this idea by highlighting the role of parental expectations in fostering traits such as prosocial behavior and conscientiousness in females, and autonomy and assertiveness in males^73^.

A larger number of close friends and a high level of group vivacity were strongly correlated with expected cyber-aggressive responses. Participants who perceived their friend groups as larger and expected more lively exchanges (texts, emoticons) in their simulated WhatsApp groups also anticipated more aggressive behavior in response to all group norms. This suggests that having more friends and frequent interactions may either simply statistically increase the likelihood of cyber-aggression or facilitate it through emotional contagion in highly active, larger groups, where emotions and behaviors, including aggression, spread from person to person^74^. This observation, while relatively new, deserves further study as it highlights the complex ways in which group dynamics influence aggressive behavior. It was crucial to control for these variables to determine the consistency of experimentally induced effects.

We did not find a relationship between Internet use and cyber-aggression, suggesting that frequent daily online activity (personal and educational use), does not increase the likelihood of expecting cyber-aggression. This finding is consistent with research by Álvarez-Garcia et al.^26^, but contrary to other studies^5,75^. It is important to note that our sample consisted of slightly older individuals and had below average daily Internet usage time^76^. Also, in contrast to Piccoli et al.^5^, we could not replicate significant association for ingroup prototypicality or ingroup identification with cyber-aggression.

Finally, we found no significant correlation between the fulfillment or subjective importance of developmental tasks and cyber-aggressive tendencies in the AYA sample. Although our approach was exploratory, this finding was unexpected given the presumed relevance of developmental milestones during this life stage^37,46,77^. The importance ratings indicated that 12 out of the 13 developmental tasks were *important* to the participants. The relatively low reliability of the measure may also partly explain the lack of significant results. The adapted DTQ-YA scale^51^, has been updated to meet current standards and developmental needs^46,42^ but was not thoroughly validated prior to our study. Future studies could explore whether specific patterns or configurations of developmental task fulfillment (e.g., establishing a career without achieving a stable partnership) are better at predicting cyber-aggression. This would require validation of the measurement instrument^78^.

This study has several limitations. Our experimental approach allowed us to investigate how group norms affect AYA's expectations of their friends’ cyber-aggression in WhatsApp groups in a controlled setting. However, our results may not fully capture the complex nature of group norms in everyday situations. The evaluation of cyber-aggression is based on hypothetical WhatsApp scenarios, which may not accurately reflect actual group dynamics and AYA reactions in real-life. We did not test if the induced group norms (funny/mocking, aggressive/insulting, or friendly/compassionate interaction tones) were perceived as intended^5^. Future studies should include a manipulation check for this issue.

Further limitations apply to the dependent variable ‘expected cyber-aggressive reactions’. To avoid social desirability and self-categorization biases, participants rated their close friends’ reactions rather than their own. While well-founded, this method has interpretability and generalizability limitations. Specifically, it assumes that individuals' perceptions of their friends' behavior reflect their own, which may not always be accurate. Research on the third-person effect suggests that individuals believe that others are more influenced by media messages than themselves, potentially skewing the data toward negative perceptions^79,80^. However, previous research (e.g., social norms approach to substance abuse prevention^81^) suggests that the tolerability of cyber-aggression within one’s own group has some impact on the respondents’ actual and future behavior. Survey research shows that quantitative, evaluative estimations are influenced by unconscious desires that reflect, at least in part, the evaluator’s cognitive style^82^.

Another limitation of the dependent variable is its simplification to a binary format. Initially, conformity to cyber-aggression was measured using multiple items (e.g., forwarding a photo, sending a nauseous emoji, writing an insulting comment) as counting variables. For practical reasons and to facilitate analysis, it was later simplified to a binary format, potentially limiting response variance. While a multilevel Poisson regression model might have been more suitable due to its ability to handle count data^83^, our study focused on testing two experimental factors and accounting for potential confounding influences, rather than making precise quantitative predictions. For purposes of the current study, distinguishing between any expected aggressive tendencies (as the lowest threshold) and no expected aggression was sufficient. However, future research should examine cyber-aggression in more detail for a more nuanced understanding. Lastly, although our research had a relatively large sample size, caution should be excercised when generalizing these results to AYA in different educational, cultural, and social contexts.

Overall, our findings indicate the importance of social dynamics, particularly group norms, in anticipating aggressive tendencies in WhatsApp groups. Although experimental effects may be smaller than in real-life situations, our results show that group norms are crucial in predicting and preventing cyber-aggression. Our analyses support a strong relationship between group norms in messaging apps and conformity behavior in cyber-aggressive scenarios. Altering these norms could reduce cyber-aggressive tendencies and increase prosocial actions^84^. As Pinho et al. argue, strategies that highlight actual group norms are essential for strengthening positive norms or reducing negative ones^84^. Our results underline the need for intervention strategies or educational trainings to help AYA develop prosocial and non-aggressive norms, fostering netiquette, and reduce negative peer-to-peer interactions. These findings could enhance current approaches^12^ and improve the current care. Our realistic WhatsApp screenshots (stimulus material) could further be used in gamified approaches that have been effective in serious games like 'Bad News' for other parts of digital competence, such as fake news detection^85^.

## Data Availability

The datasets generated during and/or analyzed during the current study are available from the corresponding author on reasonable request.
